# DeltaF508 CFTR Hetero- and Homozygous Paediatric Patients with Cystic Fibrosis Do Not Differ with Regard to Nutritional Status

**DOI:** 10.3390/nu13051402

**Published:** 2021-04-21

**Authors:** Aleksandra Mędza, Katarzyna Kaźmierska, Bartosz Wielgomas, Lucyna Konieczna, Ilona Olędzka, Agnieszka Szlagatys-Sidorkiewicz, Katarzyna Sznurkowska

**Affiliations:** 1 Department of Pediatrics, Pediatric Gastroenterology, Allergology and Nutrition, Copernicus Hospital, Nowe Ogrody 1-6, 80-803 Gdańsk, Poland; 2 Medical Clinic “Na Wzgórzu”, Jaworzniaków 37, 80-180 Gdańsk, Poland; ostasia@gmail.com; 3 Department of Toxicology, Faculty of Pharmacy, Medical University of Gdańsk, Al. J. Gen. Hallera 107, 80-416 Gdańsk, Poland; bartosz.wielgomas@gumed.edu.pl; 4 Department of Pharmaceutical Chemistry, Medical University of Gdansk, Al. Gen. J. Hallera 107, 80-416 Gdansk, Poland; lucyna.konieczna@gumed.edu.pl (L.K.); ilona@gumed.edu.pl (I.O.); 5 Department of Pediatrics, Pediatric Gastroenterology, Allergology and Nutrition, Medical University of Gdańsk, Nowe Ogrody 1-6, 80-803 Gdańsk, Poland; agnieszka.szlagatys-sidorkiewicz@gumed.edu.pl (A.S.-S.); katarzyna.sznurkowska@gumed.edu.pl (K.S.)

**Keywords:** cystic fibrosis, nutritional status, fatty acids, vitamins, body composition

## Abstract

The purpose of this study was to compare the nutritional status between deltaF508 CFTR hetero- and homozygous paediatric patients with cystic fibrosis. We assessed the percentage profiles of fatty acids measured in erythrocyte membranes and the serum levels of vitamins A, D3, E and K1 in the studied groups. We also measured the weights and heights and calculated the body mass indexes (BMIs). The studied groups consisted of 34 heterozygous and 30 homozygous patients. No statistically significant differences were found in the serum vitamins or erythrocyte membrane fatty acid profiles between the hetero- and homozygous patient groups, except for heptadecanoic acid (*p* = 0.038). The mean percentiles of height, weight and BMI did not differ significantly between the two groups. The homozygous and heterozygous paediatric patients with cystic fibrosis were similar in terms of their nutritional statuses.

## 1. Introduction

Cystic fibrosis (CF) is the most common lethal genetic disorder, affecting about 1 in 3000 people in Northern Europe [1], with a negative impact on nutrition via affecting fat and fatty acids absorption, leading to a failure to thrive and/or negative consequences for the quality of life and life expectancy.

Although the name refers specifically to fibrosis and cysts that form in the pancreas, CF is a multisystem condition that also impairs the functioning of the lungs and kidneys, as well as the hepatobiliary and gastrointestinal tracts.

CF is inherited in an autosomal recessive manner and it is a typical monogenic disease, yet it may be caused by over 2100 kinds of mutations located in the gene encoding the cystic fibrosis transmembrane conductance regulator (CFTR) protein, with more still being discovered [2].

Initially, mutations were grouped into four classes based on the molecular mechanism that affects protein synthesis and its function in different ways and with varying severities [3]. Due to the composite defects in mutant CFTR biology and pleiotropic molecular defects caused by single mutations, modifications of the current classification scheme have been proposed [4], with new classifications categorising the mutations into six [5] or even seven classes [6]. The mutations are mainly classified as either severe or mild [7], but their detection and precise molecular characterisation are crucial for the current mutation-specific treatment approaches. Currently, for many of the identified mutations, the disease liability is still unknown, and their functional consequences and clinical severity need to be determined.

Our study included patients with the most frequent mutation, which is the deletion of phenylalanine 508 in the protein delF508, which accounts for 62% of the CF cases in Poland, with 41% of the cases being homozygous, as determined by the newborn screening for cystic fibrosis program in Poland (NBS CF) [8]. The delF508 mutation was originally classified as a class II [5] mutation, but has been reclassified as class II-III-VI using the refined classifications [4]. Moreover, for the heterozygous patients included in our study, it is not an uncommon occurrence to have two different mutations of the CFTR gene that are classified differently. Thus, the severity and course of CF vary between patients, with some patients presenting only mild or atypical symptoms depending on the type of mutation. Castellani et al. noted that, in comparison to class I–III CFTR mutations, class IV and V mutations tend to be phenotypically dominant when occurring in combination with class I–III mutations, and those patients, presenting pancreatic insufficiency, have milder lung disease and live longer [9]. However, given the refined classification, the general approach may need a re-evaluation. Thus, in this study, we aimed to compare homozygotes with heterozygotes to determine the impact of the genotype on the nutritional status of paediatric CF patients.

Apart from respiratory symptoms and sweat chloride over 60 mmol/L, pancreatic exocrine insufficiency is a common clinical manifestation of CF that occurs in 80–90% of patients [10]. The consequences of pancreatic insufficiency include fat and fatty acids malabsorption and vitamin A, D3, E and K1 deficiency, as well as malnutrition, which are all frequently observed in children and adults with CF. Malnutrition in cystic fibrosis is, however, a more complex phenomenon that is related to several factors, including reduced energy intake resulting from anorexia, increased energy loss associated with gastroesophageal reflux and glucosuria in CF-related diabetes and increased energy requirements due to pulmonary destruction increasing respiratory effort [10,11,12].

Failure to thrive is a challenging aspect of CF health care. A paper concerning Polish children with CF has reported significantly delayed growth with weight and height deficits, leading to lower BMI percentiles [13]. Children with CF, especially boys, suffer more from a deficit in muscularity than from adiposity deficits. Body proportions are also affected in CF, with infantile body proportions in (older) children and abnormalities in the trunk and chest structures [14]. It has been noticed that the mutation type has a significant effect on height, weight and transverse chest diameter. Growth and weight are most significantly reduced in subjects with the non-deltaF508 genotype and are least reduced in deltaF508 heterozygous patients [13].

To the best of our knowledge, no published study has compared a profile of fatty acids measured in erythrocyte membrane and fat-soluble vitamin levels with regard to hetero- and homozygous deltaF508 CFTR mutations. Our study, measuring these parameters in combination with some anthropometric parameters and correlations between them, aimed to fill this gap.

## 2. Materials and Methods

From a group of 75 patients with a CF diagnosis, 64 children with the deltaF508 CFTR mutation were enrolled in the study. They were divided into two groups: heterozygous (*n* = 34) and homozygous (*n* = 30). All mutations in the heterozygous group are presented in Table 1. Children in our study were diagnosed based on their symptoms. None of our patients had undergone newborn screening, as the screening program had not been in place in Poland at the time they were born.

As the groups with other mutations were too small for statistical analysis, they were excluded and are not presented in Table 1.

Ages ranged from 8 to 218 months and the groups were not statistically different (*p* = 0.1).

Pancreatic insufficiency, as diagnosed via stool elastaze-1, was present in 55 individuals (85%), while 7 of the 34 heterozygotes and 2 of the 30 homozygotes were pancreatic sufficient, with no significant differences between the groups (*p* = 0.12).

None of the patients in the study received CFTR modulators. They were all treated according to CF standards, including pancreatic enzyme replacement and fat-soluble vitamin supplementation in patients with pancreatic insufficiency, as well as the correction of any nutritional deficiencies [15].

Two of our patients were on an enteral nutrition procedure using a PEG (percutaneous endoscopic gastrostomy) tube. Our lack of knowledge of the composition of their diet is a limitation of this study.

The clinical and demographic characteristics of the two groups compared in the study are shown in Table 2.

Mutations were detected using DNA sequencing in the Department of Medical Genetics, Institute of Mother and Child and Genomed S.A. Warsaw, Poland.

Blood samples were collected and serum levels of vitamin A, vitamin E, vitamin K1 and cholecalciferol (25-hydroxyvitamin D3) were determined using liquid chromatography with mass spectrometry LC-MS (product nameAgilent 1260, Agilent Technologies, Stevens Creek Blvd, CA, USA).

The percentage profiles of fatty acids that were measured in erythrocyte membranes were evaluated from venous blood using the GC-FID method [16]. Briefly, erythrocytes were lysed with water and centrifuged to separate the membranes. The pellet containing membrane, after washing with water, was extracted with chloroform/methanol (1:1). The chloroform layer was transferred into another tube and evaporated to dryness. The phospholipids were hydrolysed and methylated simultaneously in the presence of toluene and BF_3_/MeOH. Fatty acid ethyl esters (FAME) were isolated using hexane extraction. The hexane extract containing FAME was analysed on a FAME SELECT column (100 m × 0.25 mm × 0.25 µm; product name Agilent Select Fame, Agilent Technologies, Palo Alto, CA, USA) using a 456 gas chromatograph with an FID detector (Varian 3400, Walnut Creek, CA, USA). The fatty acids were expressed as the percentage of total fatty acids present in the chromatogram.

We evaluated the following anthropometric parameters: weight, height and body mass index (BMI) expressed as a BMI percentile. We used charts for weight-for-age, length/height-for-age, BMI-for-age and Z-scores according to WHO Child Growth Standards.

The Mann–Whitney *U* test and Yates’ chi-sqared test were used to verify the differences between the distributions in both groups. For the correlation analysis, we used Spearman’s test. The results were considered significant for *p* < 0.05. Statistical data processing was carried out using descriptive statistics in the Statistica 13.3 software (Statsoft, Kraków, Poland).

## 3. Results

### 3.1. Fatty Acid Profile

**Figure 1:**
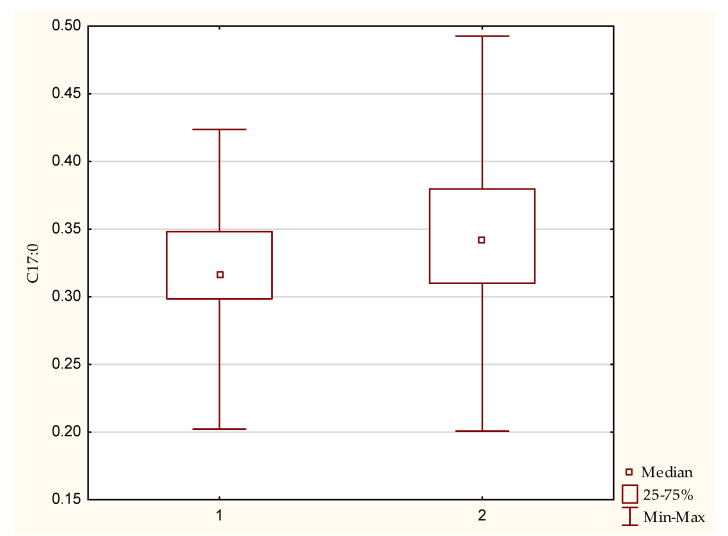
Percentage of heptadecanoic acid (C17:0) in the erythrocyte membrane in the hetero- (1) and homozygotic (2) groups, showing a statistical difference with significance *p* = 0.039.

For saturated, unsaturated and polyunsaturated fatty acids (*n*-3 and *n*-6 PUFAs) in the erythrocyte membranes, a statistically significant difference between the homozygous and heterozygous patients was only demonstrated for C17:0-heptadecanoic acid (*p* = 0.039) (Table 3), with a higher median percentage in the erythrocyte membrane in the homozygous group (Figure 1).

### 3.2. Vitamins

No statistically significant differences were found between the median serum concertations of vitamin A, vitamin E, vitamin K1 and cholecalciferol (25-hydroxyvitamin D3), as presented in Table 4.

Mean serum values indicate deficits in vitamin D3 in both groups and such deficits were in fact found in all patients in the study. With regard to vitamin E, 12 individuals (40%) were deficient in the homozygous group and 8 (23.5%) were deficient in vitamin E in the heterozygous group. No serum deficiencies in vitamin A and K1 were observed in any of the patients.

### 3.3. Anthropometric Measurements

Homozygous and heterozygous patients did not differ significantly in terms of their body mass index percentiles, height or weight, as presented in Table 5.

Most patients had a BMI between the 5th and 84th percentiles, as presented in Table 6. In the heterozygous group, two patients were over the 95th percentile.

### 3.4. Correlation Analysis

We performed a correlation analysis of the BMI percentiles with serum vitamins A, D, E and K1 levels separately in the homozygotes and heterozygotes. We did not find any correlation for vitamins with the BMI with statistical significance in the homozygotes or heterozygotes (*p* > 0.05).

We also performed a correlation analysis of the BMI percentiles with the percentages of each marked fatty acid in the erythrocyte membrane. For the homozygotes, we did not find any correlation with statistical significance (*p* > 0.05), yet for the heterozygotes, we found it for two fatty acids, as presented in Table 7.

## 4. Discussion

Although the influence of the CFTR genotype on the disease course has been studied since the discovery of the CFTR gene, this is the first study that measured differences in the serum vitamins and percentage profiles of fatty acids in the erythrocytes of F508 CFTR homozygous and heterozygous CF patients. Anthropometric parameters were included in our data to ensure a more comprehensive presentation of our patient groups.

In line with the assumption about homozygotes experiencing a more severe pattern of disease, we expected the homozygous group to present more deficits than the heterozygous group, as the literature describes this group as having a higher morbidity rate, and more often displaying pancreatic insufficiency, which could also affect their nutritional status. However, our study did not reveal such differences.

It needs to be emphasised that despite the lack of significant differences between the two groups in our study, the nutritional statuses were deficient or different from healthy controls. Prior to the current analysis, we compared almost the same group of CF patients (*n* = 75) with age-matched healthy controls (*n* = 33). Although these data have not been published, they provide a useful comparison to the general population of children in the same region of Poland; thus, we present them in Table A1 in the Appendix A. First, we noted that the mean BMI was lower in the CF patients than in the controls. Second, with regard to the fat-soluble vitamin levels, we observed that the medium serum concentration of vitamin D3, E and K1 were lower in the CF children, with no differences found for vitamin A. Finally, some differences in the percentage profile of fatty acids in the erythrocyte membranes were noted between the CF children and the healthy controls. However, the mean percentage of saturated, monounsaturated, polyunsaturated and omega-3 and omega-6 essential fatty acids were similar in the healthy controls and in the CF group, as was the omega *n*-6/*n*-3 ratio.

A different study comparing anthropometric parameters in homozygotes and heterozygotes for the deltaF508 CFTR mutation analysed weight and height percentiles and noted no significant differences [18]. Consistent with these results, our study also showed that the homo- and heterozygous groups were similar with regard to their BMI. Contrary to our data, another Polish paper reported significant differences for body height and weight, revealing that these parameters were, at a minimum, reduced in heterozygotes. Growth was reduced the most in the subjects with the deltaF508/deltaF508 and non-deltaF508 mutation genotypes [13]. This issue demands further investigation on a larger cohort, as both studies covered similar numbers of patients.

In our study, homo- and heterozygous patients did not differ in terms of the fat-soluble vitamin concentrations, which was expected due to there being similar numbers of pancreatic insufficient patients. Notably, in our study, 100% of the patients in both groups were vitamin D3 deficient (serum levels <20 ng/mL). In the literature, vitamin D3 deficiency is reported in 10–80% of patients with CF [19]. Results distinct from ours were presented in an Australian paper reporting a mean prevalence of vitamin deficiency at 15.54% for 25-OH-D3, while for other fat-soluble vitamins, the following were found: 13.13% for vitamin A, 13.89% for vitamin E and 22.62% for prolonged PT, indicating vitamin K deficiency in CF patients under 18 years old [20]. Discrepancies between our results and the quoted paper may have been caused by geographical location and the associated prevalence of vitamin deficits in the healthy population in these two countries. In Poland, vitamin D3 deficiency of various severities has been found in 90% of healthy adults, children and adolescents [21]; in Australia, on the other hand, 60% of the population is deficient [22].

We have to note that vitamin deficits are also multifactorial and do not result from pancreatic exocrine insufficiency only. For example, vitamin D3 deficiency factors include decreased sun exposure, impaired hydroxylation of vitamin D3, glucocorticoids and non-adherence to the prescribed vitamin regimen [23], which lead to decreased levels of cholecalciferol. In CF, vitamin D3 deficiency has been observed despite daily supplementation [24], which is consistent with our results, as we also noted deficits in all patients who received vitamin D3 supplementation in both groups. Although our patients received vitamin supplementation, the lack of information on specific doses for each individual is one of the limitations of this study.

The fatty acids profile in CF has been widely described in the literature and is mostly based on fatty acids serum levels [25,26,27,28,29,30] and/or erythrocyte membrane or other tissue profiles [31,32,33,34].

Christoph et al. demonstrated the differences in the serum profile of fatty acids between homo- and heterozygotes, with a relative essential fatty acid deficiency found in CF patients compared with the controls and with less pronounced differences noted for heterozygotes than homozygous patients [35]. However, the specific meaning of the homo- or heterozygous was not clarified by the authors and it was not specified whether it referred to the F508 CFTR mutation or a group of other mutations.

Others investigated fatty acid profiles, both in serum and in the erythrocyte membrane, but compared patients depending on their pancreatic function. These results cannot be extrapolated to our data, as in our study, both groups largely displayed pancreatic insufficiency. Interestingly, the study concluded that pancreatic insufficiency and sex influenced fatty acids in plasma and erythrocytes [31].

Patients in both groups in our study were not supplemented with either PUFAs or other fatty acids. As PUFAs cannot be synthesised in the human body, they must be obtained from one’s diet. Although the consumption of fatty acids increases their serum concentration [36], its supplementation is still controversial in CF patients due to poor clinical effects on the disorder course [37,38].

The only difference in the fatty acids profile in the erythrocyte membrane revealed in our study concerned heptadecanoic acid. We have not found any other paper describing such a difference in the fatty acids profile in the available databases. One must note that we did not compare the total amounts of particular fatty acids but their ratio in the erythrocyte membrane.

Published data suggest that metabolic differences may lead to essential fatty acids deficiencies depending on the type of CFTR mutation. It has been reported that serum concentrations of linoleic acid and docosahexaenoic acid were significantly lower in patients who had severe CF transmembrane conductance regulator mutation (homozygotes for deltaF508 and heterozygotes/homozygotes for 394delTT) than the other groups (e.g., heterozygotes for deltaF508 CFTR mutation), excluding the impact of pancreatic insufficiency [30]. However, the data concerned serum concentrations, not the fatty acids profile in erythrocyte membranes.

Heptadecanoic acid (C17:0) has been a subject of increasing research interest in relation to health and some diseases, especially type 2 diabetes and coronary heart disease. After hydrolysis from triheptadecanoic acid, this acid was utilised to develop the Malabsorption Blood Test (MBT), which assesses fat malabsorption in patients with cystic fibrosis and pancreatic insufficiency [39]. However, others claim that the plasma concentration of heptadecanoic acid is not a precise biomarker of dairy fat intake [40], indicating it can also be synthesised endogenously, e.g., from gut-derived propionic acid (3:0). One may thus assume that dairy-derived heptadecanoic acid can increase endogenously in body tissues, and, relevant to our study, on the RBC membrane.

Due to its possible biochemical transformation pathways, heptadecanoic acid can replenish the citric acid cycle with anaplerotic intermediates and improve mitochondrial energy metabolism. This helps to support oxidative balance, especially when metabolic stress increases [41]. It is still unknown why the percentage of heptadecanoic acid on the erythrocyte membrane in homozygotes is higher than in heterozygotes and the answer remains to be found.

We decided to analyse erythrocyte membrane fatty acids, as they may serve as a biomarker of fatty acid dietary levels and fats absorption over several months as the circulation half-life time of erythrocytes exceeds 100 days [42]. Moreover, erythrocyte membrane lipids may better reflect the fatty acid composition of tissues than plasma lipid levels, which indicates the day-to-day consumption and absorption and thus offers a less stable indicator.

We expected some differences in the erythrocyte membrane profiles between the groups, as supported by studies reporting that EFA deficiency in CF is caused by a defective regulation of EFA metabolism. The deltaF508 mutation in the CFTR alters the control of essential fatty acid utilisation in pancreatic epithelial cells [43]. Further study is needed in a larger cohort in specific age categories. Although our cohort consisted of only paediatric patients, the age ranged widely from 8 up to 218 months. Reference data for the fatty acid composition of erythrocyte membrane lipids in children is rather limited, but it has been reported that the profile is age-dependent in healthy children [44,45]; thus, the above mention of our unpublished results presenting the fatty acids profile in healthy Polish children.

Although CF is a monogenic disorder, one must note that the genotype–phenotype link in CF is complex. In addition to the type of genetic mutation, the severity of CF is also determined either by the context in which the defective gene operates (i.e., genetic background) or environmental influences [46]. Nevertheless, certain conclusions can be derived from studies reflecting the clinical diversity in patients who are homo- and heterozygous for the deltaF508 mutation.

Even though the assays in our study did not show diversity in heterozygotes with regard to the analysed parameters, as they were similar to the homozygotes, we admit that the heterozygotes comprised a heterogeneous group. In the 34 individuals constituting the heterozygous group, we detected 24 kinds of mutations other than deltaF508, some occurring more than once. This may motivate further study to compare exactly the same heterozygous patients with homozygotes. The numbers for specific mutations were too low to perform a separate statistical analysis. Some mutations belonged to rare or unknown classes and several CFTR mutations cause multiple functional consequences, and hence, cannot be assigned to one particular class. Moreover, the categorisation of CFTR mutations in classes is a research tool and is not predictive of clinical outcomes in individual patients [9]. Thus, we do not provide data on the classification of other mutations we found.

Unexpectedly, in our study, the majority of patients in both groups presented pancreatic insufficiency with no statistically significant differences between the homo- and heterozygous groups. This finding is inconsistent with a commonly known fact that patients who are homozygous for the deltaF508 mutation have a higher incidence of pancreatic insufficiency. Heterozygous patients tend to be older at the time of diagnosis and present with a milder disease [18]. Another clinical aspect of CF presentation in homo- and heterozygous patients was examined by Johansen et al., who provided data about poorer lung function in homozygous patients, with the calculated yearly incidence of chronic Pseudomonas aeruginosa infection and yearly mortality rates being greater in homozygous than in heterozygous patients [47]. Contrary to our results, the quoted paper demonstrated clinical diversity between these two groups of CF patients.

To find an association between the deficit parameters, we correlated the BMI percentiles with the serum levels of vitamins A, D3, E and K1 and the BMI percentiles with fatty acids in the erythrocyte membranes. From 68 correlations, we found only two that were statistically significant, which were in the heterozygotic group between BMI percentiles and fatty acids. This finding demands a further investigation on a larger cohort with respect to the individual daily diet and supplementation, as the reason for these correlations remains unknown.

## 5. Conclusions

Overall, according to our data, nutritional parameters, such as fat-soluble vitamins, fatty acid profile, weight, height and BMI, did not differ between the groups of paediatric hetero- and homozygous patients for the deltaF508 CFTR mutation. We may thus conclude that the genotype in cystic fibrosis did not accurately predict the individual nutritional phenotype. All cystic fibrosis patients, irrespective of their type of mutation, should be placed under individual nutritional management as soon as possible and treated to prevent further consequences of malnutrition or failure to thrive. The fat-soluble vitamin deficits found in our cohort, despite supplementation, indicated that CF patients were at a high risk of fat-soluble vitamin deficiencies and targeted supplementation might be required. Regular vitamin D3 serum level testing would be beneficial to determine the adequate doses of supplementation to suit the increased demand.

## Figures and Tables

**Table 1:** Types of mutation detected in the heterozygous subgroup for deltaF508.

Type of Mutation	Number of Patients	Percentage
dele2,3(21 kb)	5	14.7
1717–1G > A	3	8.82
3659 delC	2	5.88
3600 + 2 insT	2	5.88
N1303K	2	5.88
3849 + 10 kbC > T	2	5.88
4374 + 1G > T	1	2.94
3171insC	1	2.94
IVS2 + 1G > T	1	2.94
N1282X	1	2.94
E92K	1	2.94
R1158X	1	2.94
2184insA	1	2.94
K710X	1	2.94
dup16,17a	1	2.94
G542X	1	2.94
1078delT	1	2.94
M952T	1	2.94
2184insA	1	2.94
Y1092X	1	2.94
296 + 1G > T	1	2.94
IVS-5T(TG)11	1	2.94
3272–26A-G	1	2.94
2143delT	1	2.94

**Table 2:** Clinical and demographic features of the subjects in the homozygous and heterozygous groups.

Clinical and Demographic Features	Homozygotes	Heterozygotes	*p*
Total number	30	34	-
Mean age (months) (range)	116 (10–198)	92.1 (8–216)	0.1
Sex: females *n* (%)	16 (53.3)	15 (44.1)	0.96
Sex: males *n* (%)	14 (46.7)	19 (55.9)	0.96
Mean stool elastaze-1 (μg/g) ^1^ (range)	86.7 (15–200)	147.3 (16–652)	0.37
*Pseudomonas aeruginosa*-colonised patients *n* (%)	6 (20)	8 (23.5)	0.96

^1^ Due to laboratory limitations, if the results were marked <15 μg/g, we assumed 15 μg/g for statistical calculations.

**Table 3:** Mean percentage of fatty acids in the erythrocyte membrane in the homozygous and heterozygous groups.

Fatty Acid (%)		Homozygous	Heterozygous	*p*
Saturated	C 14:0	0.58	0.58	0.95
	C 15:0	0.17	0.16	0.94
	C 16:0	25.12	25.08	0.85
	C 17:0	0.34	0.32	0.04
	C 18:0	15.45	15.95	0.55
	C 20:0	0.49	0.50	0.81
	C 22:0	1.61	1.58	0.82
	C 24:0	4.01	4.03	0.94
Unsaturated	C16:1 *n*-7 cis	0.38	0.34	0.39
	C17:1	0.09	0.08	0.27
	C18:1 *n*-9 trans	0.16	0.15	0.68
	C18:1 *n*-7 trans	0.20	0.20	0.56
	C18:1 *n*-9 cis	12.03	11.87	0.55
	C18:1 *n*-7 cis	0.98	0.94	0.61
	C20:1 *n*-9 cis	0.35	0.62	0.51
	C22:1	0.24	0.20	0.48
	C22:1 *n*-9 cis	0.23	0.25	0.24
	C24:1 *n*-9	5.36	5.22	0.45
Polyunsaturated omega *n*-3	C16:3 *n*-3	0.09	0.10	0.53
	C18:3 *n*-3	0.21	0.20	0.67
	C20:5 *n*-3	1.46	1.36	0.34
	C22:5 *n*-3	2.31	2.24	0.95
	C22:6 *n*-3	3.69	3.57	0.81
Polyunsaturated omega *n*-6	C18:2 *n*-6 cis	7.83	7.95	0.88
	C18:3 *n*-6	0.08	0.07	0.10
	C20:2	0.26	0.24	0.58
	C20:3 *n*-6	1.70	1.62	0.98
	C20:4 *n*-6	11.62	11.62	0.99
	C22:2	0.10	0.09	0.98
	C22:4 *n*-6	2.86	2.86	0.99

**Table 4:** Mean percentages of the serum levels of vitamin A, vitamin E, vitamin K1 and cholecalciferol (25-hydroxyvitamin D3) in the homozygous and heterozygous groups.

Vitamin	Mean Serum Levels in Heterozygotes ± SD (Range)	Mean Serum Levels in Homozygotes ± SD (Range)	*p*	Reference Value ^2^
A (μg/mL)	0.78 ± 0.2 (0.47–1.18)	0.77 ± 0.18 (0.44–1)	0.73	0.11–0.97
25-OH-D3 (ng/mL)	12.92 ± 3.41 (8.53–17.91)	13.14 ± 3.31 (7.18–18.02)	0.82	20–50
E (μg/mL)	6.32 ± 3.49 (0.75–13.21)	6.50 ± 3.39 (0.72–11.97)	0.80	3.8–18.4
K1 (ng/mL) ^1^	0.41 ± 0.21 (0.18–0.7)	0.38 ± 0.2 (0.18–0.67)	0.38	0.10–2.20

^1^ Due to laboratory limitations, if the results were marked <0.181 ng/mL, we assumed 0.18 ng/mL for the statistical calculations. In the homozygotes, there were 13 such patients and 12 in the heterozygotes. ^2^ According to the Mayo Clinic Laboratory reference values [17].

**Table 5:** Anthropometric indicators by genotype group.

Anthropometric Indicators	Homozygotes (*n* = 30)	Heterozygotes (*n* = 34)	*p*
Mean height percentile (range)	41.72 ± 33.75 (0.2 to 95.5)	49.24 ± 31.4 (2.6 to 99.8)	0.3
Mean height Z-score (range)	−0.40 ± 1.28 (−2.96 to 1.69)	0.07 ± 1.2 (−1.94 to 2.9)	0.3
Mean weight percentile (%)	35.74 ± 28.67 (0.2 to 84.9)	49.65 ± 34.43 (0.2 to 99.2)	0.11
Mean weight Z-score (range)	−0.58 ± 1.07 (−2.84 to 1.03)	−0.06 ± 1.32 (−2.9 to 2.4)	0.11
Mean BMI (range)	16.57 ± 2.12 (13.6 to 21.0)	16.97 ± 2.3 (13.8 to 21.8)	0.48
Mean BMI percentile (range)	36.07 ± 26.09 (0.7 to 86.0)	48.73 ± 31.48 (0.2 to 97.6)	0.11
Mean BMI percentile Z-score (range ± SD)	−0.5 ± 0.9 (−2.48 to 1.11)	−0.03 ± 1.1 (−2.82 to 1.97)	0.11

BMI: Body Mass Index.

**Table 6:** Number of individuals in the BMI percentile ranges in the homozygous and heterozygous groups.

BMI Percentile	Homozygotes *n* = 30	Heterozygotes *n* = 34
<5	10% (*n* = 3)	5.9% (*n* = 2)
5–24	23.3% (*n* = 7)	23.5% (*n* = 8)
25–84	63.3% (*n* = 19)	50% (*n* = 17)
85–94	3.3% (*n* = 1)	14.7% (*n* = 5)
≥95	0% (*n* = 0)	5.9% (*n* = 2)

**Table 7:** Select data on the correlations of BMI with fatty acids in the erythrocyte membrane that had statistically significant differences.

Correlated Parameters	*n*	Spearman’s *R*	*p*
BMI Percentile with α-linolenic acid (C18:3 *n*-3)	32	−0.37	0.04
BMI Percentile with heptadecenoic acid (C17:1)	32	0.48	0.00

## Data Availability

The data presented in this study are available on request from the corresponding author.

## Appendix A

Table A1 presents a comparison between paediatric patients with CF (*n* = 75) and healthy children (*n* = 33) aged 9–216 months who received treatment in an orthopaedic or endoscopic unit. Physical examination and blood samples were collected before anaesthesia due to corrective surgery or the removal of a foreign body from the stomach. Patients from the control group did not suffer from any chronic disease.

**Table A1:** Comparison of nutritional status between healthy children and CF patients.

Parameters	CF Patients *n* = 75	Healthy Controls *n* = 33	*p*
Mean age (months) (range)	110.3 ± 64.6 (9–216)	116 ± 65 (9–216)	*p* = 0.71
Anthropometric Measurements			
Mean FFM%-anthropometric	77.53 ± 6.54	68.77 ± 9.4	*p* = 0.00
Mean FM%-anthropometric	22.47 ± 6.54	31.23 ± 9.4	*p* = 0.00
Mean FFM%-BIA	81.20 ± 10.93	80.77 ± 8.06	*p* = 0.08
Mean FM%-BIA	17.6 ± 6.04	19.23 ± 8.06	*p* = 0.08
Mean BMI	16.8 ± 2.2	19 ± 4.00	*p* = 0.02
BMI Percentile			
<3	8%	3.03%	-
3–15	12%	3.03%	-
15–50	45.3%	36.4%	-
50–85	24%	24.24%	-
85–97	9%	18.18%	-
≥97	2.7%	15.15%	-
Fat-Soluble Vitamins Mean			
A (μg/mL)	0.8 ± 0.2	1.0 ± 0	*p* = 0.09
25-OH-D3 (ng/mL)	13.0 ± 3.3	24.0 ± 5.0	*p* = 0.0
E (μg/mL)	6.1 ± 3.4	10.0 ± 2.0	*p* = 0.0
K1 (ng/mL)^2^	0.4 ± 0.2	1.0 ± 0.0	*p* = 0.03
Select Data on Fatty Acid Profiles in Erythrocyte Membranes: Acids with Statistically Significant Differences			
Saturated			
Myristic acid (C14:0)	0.6 ± 0.2	0.0 ± 0	*p* = 0.0
Lignoceric acid (C24:0)	4.0 ± 0.6	4.0 ± 0	*p* = 0.001
Behenic acid (C22:0)	1.6 ± 0.3	2.0 ± 0	*p* = 0.001
Monounsaturated			
Palmitoleic acid (C16:1 *n*-7)	0.4 ± 0.2	0.0 ± 0	*p* = 0.0
Heptadecenoic acid (C17:1)	0.1 ± 0	0.0 ± 0	*p* = 0.04
Vaccenic acid (C18:1 *n*-7 cis)	1.0 ± 0.2	1.0 ± 0	*p* = 0.038
Erucic acid (C22:1)	0.2 ± 0.1	0.0 ± 0	*p* = 0.009
PUFA: Omega *n*-6			
Gamma-linolenic acid (18:3 *n*-6)	0.1 ± 0.0	0.0 ± 0	*p* = 0.00
Dihomo-gamma-linolenic acid (20:3 *n*-6)	1.7 ± 0.4	1.0 ± 0	*p* = 0.003
Linoleic acid (18:2 *n*-6)	7.9 ± 1.5	9.0 ± 1	*p* = 0.0
PUFA: Omega *n*-3			
Hexadecatrienoic acid (16:3 *n*-3)	0.1 ± 0.1	0.0 ± 1	*p* = 0.024
